# Indiana Dental College

**Published:** 1884-08

**Authors:** 


### Indiana Dental College.

In a circular just received from the secretary of this institution is the following announcement: "The Trustees of this College have ordered that henceforth their Faculty shall require two full terms as a requisite for possible graduation, provided, however, that five years practice, with satisfactory preliminary examination upon practical dentistry, may be accepted as equivalent to one course at Dental College." This is a step in the right direction, and will be gratifying to all earnest friends of dental education. But it should, ere long, be followed by other steps, for instance: dropping the five years practice as equal to one course in college; requiring a preliminary examination; extending the length of term. These are suggestions not for this college only, but for any who may be inclined to adopt them. Our earnest desire is that every college should do the most thorough work, and that the competition should be rather in this direction, than in the effort to secure the largest number of students.

